# Supplementary material for: Cost-effectiveness of human T-cell leukemia virus type 1 (HTLV-1) antenatal screening for prevention of mother-to-child transmission
Source: PLoS Negl Trop Dis. 2023 Feb 21;17(2):e0011129. doi: 10.1371/journal.pntd.0011129 (PMC9983854; doi:10.1371/journal.pntd.0011129)
Supplement: Attachment — Submitted filename: Response to Reviewers comments.docx [file pntd.0011129.s001.docx]

Responses to reviewer comments

Thank you very much for the reviewers’ very important comments. According to very important comments, I completely revised my paper point by point and highlighted my responses to the reviewers’ comments in red. I have written the page and line numbers of the highlighted version of the text to indicate where changes were made.

My deepest thanks to the reviewers.

Sincerely yours,

Akiko Kowada, MD, PhD

Reviewer #1: The addition of a cost effectiveness study from Japan on antenatal screening for prevention of mother-to-child HTLV-1 transmission is welcome. Indeed, it is interesting that no such study has previously been published either leading up to the decision to extend HTLV antenatal screening nationally circa 2011 or since. The only previous study, from the UK– a low prevalence country – concluding that even in the UK screening could be cost effective(1). The paper by A. Kowada concludes that antenatal screening in Japan meets the cost criteria for screening based on provider willingness to pay values of US$ 50,000 or 100,000 per QALY. These are higher than in the UK.

Major Comments.

1. The life-time risk of HAM/TSP cited in the introduction is 0.18 – 1.8% (although higher rates have been reported. Which rate was used for this study?

Response) Thank you very much for your important comments. I didn’t use the rates in the model from this sentence “The lifetime risk in HTLV-1 infected individuals is estimated to be 5% for ATL and 0.18-1.8% for HAM/TSP.”, so I deleted this sentence (Page 7, line 126-127).

I used the annual HAM/TSP rate, 0.000033, in the model from the literature (Ref. Japanese Society of Neurology. JSN Practical guidelines for HTLV-1-associated myelopathy (HAM), 2019.　[Japanese] https://www.neurology-jp.org/guidelinem/ham/ham_2019.pdf).

2. The life-time risk of ATL is cited in the introduction to be 5%. Was the association of ATL risk with acquisition of HTLV-1 in early life considered in the analysis?

Infection early in life is associated with a higher risk of disease development. Adult T-cell leukemia (ATL), which is caused by HTLV-1 and has a median survival of 8 months is linked to mother-to-child transmission (MTCT), indeed evidence of ATL following infection as an adult is sparse. Infective dermatitis also only occurs following neonatal infection.

Response) Thank you very much. I didn’t use this estimated lifetime risk in the model. So, I deleted the sentence, “The lifetime risk in HTLV-1 infected individuals is estimated to be 5% for ATL.” (Page 7, line 126-127). I used the annual ATL rate, 0.000976, in the model from the literature (Ref. Arisawa K, Soda M, Endo S, Kurokawa K, Katamine S, Shimokawa I, et al. Evaluation of adult T-cell leukemia/lymphoma incidence and its impact on non-Hodgkin lymphoma incidence in southwestern Japan. Int J Cancer. 2000;85:319-324.).

　Yes, the association of ATL risk with acquisition of HTLV-1 in early life was considered in the analysis. In the model, HTLV-1 is transmitted mainly through mother-to-child transmission (MTCT), even if horizontal transmission through sexual intercourse is being considered.

3. Why were the thresholds $50 and $100,000 chosen? What is the accepted cost per QALY in Japan?

Response) In Japan, two WTP thresholds, US$50,000 and US$100,000 per QALY gained, are generally used in cost-effectiveness analysis (Ref. Iino H, Hashiguchi M, Hori S. Estimating the range of incremental cost-effectiveness thresholds for healthcare based on willingness to pay and GDP per capita: A systematic review. PLoS One. 2022 Apr 14;17(4):e0266934. doi: 10.1371/journal.pone.0266934. PMID: 35421181; PMCID: PMC9009631.

Ref. Ministry of Health, Labor and Welfare. https://www.mhlw.go.jp/file/05-Shingikai-12404000-Hokenkyoku-Iryouka/0000211609.pdf (Japanese) Accessed 20 October 2022.).

However, I undestood the reviewer's comments and reset the WTP threshold to $50,000 per QALY gained in this study because the ICER, US$40,318 per QALY gained, was lower than $50,000/QALY gained.

Page 3, line 50. In the abstract, I added the sentence, “The willingness-to-pay threshold was set at US$50,000 per QALY gained.”.

Page 10, line 171-173. I revised the sentence to “Incremental cost-effectiveness ratios (ICERs) were calculated and compared to a willingness-to-pay (WTP) threshold of US$50,000 per quality-adjusted life-year (QALY) gained”.

4. In the methods it is not clear what “other causes” of mortality were considered in the model. In the results on HAM/TSP and ATL cases are presented. Please clarify. Also, what impact does the adj Mortality Rate of 1.57 as reported by Schierhout(2) have on the cost effectiveness? Noting that this cannot be attributed to deaths from ATL or HAM.

Response) Thank you very much.

Page 13, line 231-233. I added the impact of the adj Mortality Rate of 1.57 to the mortality from other causes in the model and added the sentence, “The mortality from other causes was calculated by the adjusted risk of death due to any cause in people with HTLV-1 infection when compared with HTLV-1-negative counterparts and the values obtained from vital statistics (Ref. Schierhout G, McGregor S, Gessain A, Einsiedel L, Martinello M, Kaldor J. Association between HTLV-1 infection and adverse health outcomes: a systematic review and meta-analysis of epidemiological studies. Lancet Infect Dis. 2020;20(1):133-143. doi:10.1016/S1473-3099(19)30402-5).” in the methods.

5. In the model structure the assumption is that all mothers breast-feed. Is this the case in Japan and if so for how long?

Response) Thank you very much for your great comments. I have changed the assumption based on the literature (Ref. Ministry of Health, Labor and Welfare. Infant and Young Child Nutrition Survey. 2015.https://www.mhlw.go.jp/file/06-Seisakujouhou-11900000-Koyoukintoujidoukateikyoku/0000134207.pdf. (Japanese) Accessed 20 October, 2022. Ref. Inano, H, Kameya, M, Sasano, K, Matsumura, K, Tsuchida, A, Hamazaki, K, et al. Factors influencing exclusive breastfeeding rates until 6 months postpartum: the Japan Environment and Children's Study. Sci Rep. 2021;11:6841.). In the new model, the proportion of long-term breast-feeding* (≧6 months) is 37.4%, the proportion of short-term breast-feeding (＜6 months) is 59.1%, and the proportion of bottle feeding is 3.5%. Please see Table 1.

6. What is the negative impact (QALY cost) of not breast-feeding?

Response) There are two main negative impacts of not breastfeeding. For mothers, it increases the long-term risk of cancer (breast, ovarian, endometrium), endometriosis, diabetes, osteoporosis, hypertension, cardiovascular diseases, metabolic syndrome, rheumatoid arthritis, Alzheimer disease, multiple sclerosis. For infants, it increases the risk of otitis media, upper and lower respiratory tract infection, asthma, RSV bronchiolitis, atopic dermatitis, gastroenteritis, inflammatory bowel disease, diabetes, leukemia, and sudden infant death syndrome.

Page 32-33, line 406-412. I added these negative impacts of not breast-feeding in the discussion.

Ref. Del Ciampo LA, Del Ciampo IRL. Breastfeeding and the Benefits of Lactation for Women's Health. Aleitamento materno e seus benefícios para a saúde da mulher. Rev Bras Ginecol Obstet. 2018;40(6):354-359. doi:10.1055/s-0038-1657766

Ref. American Academy of Pediatrics, Section on Breastfeeding. Breastfeeding and the use of human milk. Pediatrics. 2012;129(3):e827-e841. doi:10.1542/peds.2011-3552

Page 33, line 424-426. The sentence, “Sixth, the adverse health effects on mothers and infants of withholding breastfeeding were not considered in the model.” was also added in the limitations.

7. The effectiveness of the intervention is given as reducing transmission from 20% to 3% but the rate of transmission is determined by the duration of breast feeding. This should be taken into account in the model.

Response) Thank you so much for the important comment.

In the new model, I have considered three different HTLV-1 transmission rates (HTLV-1 transmission rate with long-term breast-feeding (≧6 months)=0.203, HTLV-1 transmission rate with short-term breast-feeding (＜6 months)=0.074, and HTLV-1 transmission rate with bottle feeding=0.025). Please see Table 1.

Ref. Hino S. Establishment of the milk-borne transmission as a key factor for the peculiar endemicity of human T-lymphotropic virus type 1 (HTLV-1): the ATL Prevention Program Nagasaki. Proc Jpn Acad Ser B Phys Biol Sci. 2011;87:152-166.

8. The cost of the antibody screening is given as US$ 76.4 per person inclusive of confirmatory test. How was this calculated? It is taken from a table of medical fees but high-volume screening is likely to be cheaper in terms of the reagents and in personnel as it is added to other screening assays. Has this been considered?

Response) Page 16, line 244-249. The cost of the HTLV-1 antibody test was calculated based on the Japanese national fee schedule and medical insurance reimbursement table. The cost consisted of the antibody test by chemiluminescence immunoassay (CLIA)　method, the confirmation test by line-blot assay (LIA) method, and the immunological test decision fee. I added the explanation in the methods. I have modified and changed some of the methods for HTLV-1 antibody testing in this model.

Thank you so much for your great suggestion.

Page 29, line 346-349. I did not consider the lower cost of the screening test through high-volume screening. I added the sentence, “For 10,517,942 individuals born between 2011 and 2021, HTLV-1 antenatal screening cost US$787 million more than no screening. However, further cost savings may be expected in the future by promoting low-cost HTLV-1 antenatal screening through high-volume screening worldwide.” to the discussion because of the potential for future cost savings.

9. The impact of sexual transmission was considered. What was estimate of life-time risk that a person infected through in early life would transmit HTLV-1 through sexual intercourse? How was it calculated? Was this different for males and females?

Response) I estimated the lifetime risk of sexual transmission by the Markov cohort model using the rate from the literature (Satake M, Iwanaga M, Sagara Y, Watanabe T, Okuma K, Hamaguchi I. Incidence of human T-lymphotropic virus 1 infection in adolescent and adult blood donors in Japan: a nationwide retrospective cohort analysis. Lancet Infect Dis. 2016;16:1246-54.). Please see Table 1.

There is a gender difference in HTLV-1 horizontal infection rates.

Page 13, line 229-231. I added the sentence, “The horizontal HTLV-1 infection rate was based on the average of men and women from a nationwide retrospective cohort analysis.” in the methods.

Page 33, line 417-420. I also revised the sentence in the limitations, “Third, gender differences in HTLV-1 horizontal transmission rates were not taken into account in the model. The HTLV-1 horizontal transmission rate from males to females is greater than the HTLV-1 horizontal transmission rate from females to males.”.

Ref. Satake M, Iwanaga M, Sagara Y, Watanabe T, Okuma K, Hamaguchi I. Incidence of human T-lymphotropic virus 1 infection in adolescent and adult blood donors in Japan: a nationwide retrospective cohort analysis. Lancet Infect Dis. 2016;16:1246-54.

10. In the section on cumulative lifetime health effects the number of cases of ATL and HAM prevented seem small for ATL with an expected ATL rate of 2.8% only and very small for HAM with a lifetime risk of just 0.15%. A detailed explanation of these rates is required for the reader to understand the findings.

Response) Thank you very much for the important comments.

Page 26-27, line 336-338. I added the sentence, “The number of ATL and HAM/TSP losses is not equal to the number of HTLV-1 carriers’ losses because the incidence of ATL and HAM/TSP is too low.” for the explanation in the results.

11. In the analysis how are twins accounted for?

Response) Twins were not accounted for in the analysis.

Page 33, 432. I added the sentence, “Eighth, twins were not taken into account in the analysis.” in the limitations.

12. In Figure 2C the pivot point for effectiveness seems to be at about $25,000 per QALY). Why consider $100,000 per QALY?

Response) Thank you so much for the important comment. I recalculated the cost-effectiveness results in the new model and got the ICER, US$40,318 per QALY gained, in the base-case analysis. I followed the reviewer's comments and reset the WTP threshold to $50,000 per QALY gained because the ICER was lower than $50,000/QALY gained in this study.

Page 3, line 50. “The willingness-to-pay threshold was set at US$50,000 per QALY gained.”

13. Table 3 is confusing with cumulative life-time cost of HTLV-1 antenatal screening given as US$ 866 million and various sums presented as reduction regardless of whether these are losses or gains.

Response) Thank you very much for the significant comment. To resolve this confusion, I have added two new rows, “Loss” and “Gain” to Table 3.　Please see Table 3.

14. Furthermore the reduction in cases in Table 3 requires explanation. Why is the reduction in disease not at the same as the reduction in cases of infection?

Response) Thank you very much.

Page 26-27, line 336-338. I added the sentence, “The number of ATL and HAM/TSP losses is not equal to the number of HTLV-1 carriers’ losses because the incidence of ATL and HAM/TSP is too low” to the results.

15. In the discussion reference is made to the implementation of a national antenatal screening programme in 2011 and that recently maternal seroprevalence has been decreasing. Clearly an antenatal screening programme from 2011 cannot have impacted maternal seroprevalence already especially as the average age of mothers in Japan is 30 years. Comment should be made on the extent of screening in Japan in the preceding years noting the introduction of HTLV antenatal screening in Nagasaki was early as the late 1980s?

Response) Thank you very much for the significant comments. Page 8, line 140-145. I added the sentences, “Laboratory screening for HTLV-1 infection has been routine practice for blood donors since 1986. The ATL Prevention Program in Nagasaki from 1987 to 2004 revealed a marked reduction of HTLV-1 mother-to-child transmission by withholding breast-feeding for carrier mothers and has recommended avoiding breastfeeding as the most reliable method for mother-to-child transmission prevention.” in the introduction.

Ref. Itabashi K, Miyazawa T, Sekizawa A, et al. A Nationwide Antenatal Human T-Cell Leukemia Virus Type-1 Antibody Screening in Japan. Front Microbiol. 2020;11:595. Published 2020 Apr 9. doi:10.3389/fmicb.2020.00595

16. In the comment on depression and anxiety whilst acknowledging that all diagnostic tests and screening programmes are inevitably associated with a degree of anxiety the current statement implies that this would be particularly a feature of HTLV screening and does not take into account the depression and anxiety that occurs when parents discover that they were not offered testing and interventions for a preventable infection causing cancer and neurological disease (as HTLV-1) and see these occurring in the children (even as adults). It is important to discuss both elements since the psychological aspects are not included – not just the potential negative aspects.

Response) Thank you very much for the important comments. Page 32, line 394-401. I added the sentence in the discussion, “All diagnostic tests and screening programmes are inevitably associated with a degree of anxiety. This would be particularly a feature of HTLV-1 antenatal screening. Depression and anxiety would occur when parents discover that they were not offered testing and interventions for a preventable infection causing cancers and progressive neurological diseases and see these diseases occurring in their children even as adults. When introducing HTLV-1 antenatal screening, it is necessary to consider an enhanced counseling system to minimize the negative psychological aspects of HTLV-1 carriers.”

1. Malik B, Taylor GP. Can we reduce the incidence of adult T-cell leukaemia/lymphoma? Cost-effectiveness of human T-lymphotropic virus type 1 (HTLV-1) antenatal screening in the United Kingdom. Br J Haematol. 2018;184:1040-3.

2. Schierhout GM, S; Gessain, A; Einsiedel, L; Martinello, M, Kaldor, J. The association between HTLV-1 infection and adverse health outcomes:a systematic review and meta-analysis of epidemiologic studies. Lancet Infect Dis. 2019.

3. de The G, Bomford R. An HTLV-I vaccine: why, how, for whom? AIDS Res Hum Retrovirol. 1993;9(5):381-6.

4. Gessain A, Cassar O. Epidemiological Aspects and World Distribution of HTLV-1 Infection. Front Microbiol. 2012;3:388.

Response) Thank you very much for introducing four important references. I have added and cited all references in the manuscript.

1. Page 29, line 352-354. Malik et al suggested, based on a modelling study, that HTLV-1 antenatal screening meets the cost-efficacy standards in the UK, one of HTLV-1 low-prevalence countries [38].
2. Page 13, line 231-233. The mortality from other causes was calculated by considering adjusted risk of death due to any cause in people with HTLV-1 infection when compared with HTLV-1-negative counterparts [28]
3. Page 31, line 383-391. However, HTLV-I causes serious diseases, ATL and HAM/TSP, and thus places in the same category of viruses for which efficient vaccines are made and used. Furthermore, there are factors favoring the feasibility of the vaccine against HTLV-I: HTLV-1 has a relatively low antigenic variability, natural immunity occurs in humans, and experimental vaccination with the envelope antigen is successful in animal models. Vaccines against HTLV-I would have important public health value in oncology, neurology, and AIDS, and are expected to play a pioneering role in the field of oncology, neurology, and AIDS [46].
4. Page 7, line 119-121. An estimated 5 to 10 million people worldwide are infected with HTLV-1 although data are missing or sparse for up to 6/7th of the global population, based on published seroprevalence rates [5,6]

Minor Comments.

1. The estimate of 20 million people infected with HTLV-1 worldwide is from de The & Bomford(3), not Gessain & Cassar(4)– who estimated 5 – 10 million based on published seroprevalence but acknowledged that data pertinent to 6/7th of the global population were missing or sparse.

Response)　Thank you very much for very important comments.

I corrected the sentence, “Based on published seroprevalence rates, which are missing or sparse for up to 6/7th of the global population, an estimated 5 to 10 million people worldwide are infected with HTLV-1.” (Page 5, line 82-84) in the author summary and “An estimated 5 to 10 million people worldwide are infected with HTLV-1 although data are missing or sparse for up to 6/7th of the global population, based on published seroprevalence rates.” (Page 7, line 119-121) in the introduction.

2. Regions of high prevalence include Iran and Central Australia – this is mentioned in the discussion but omitted from the introduction.

Response) Thank you very much. I added the regions of high prevalence including Iran and Central Australia in the introduction.

Page 7, line 121-124. “The regions of HTLV-1 high prevalence are limited to the Southwestern part of Japan, sub-Saharan Africa and South America, the Caribbean area, and foci in the Middle East and Australia-Melanesia.”.

3. In the author summary an explanation of the type of disease would be helpful for HAM/TSP (as is given for ATL).

Response) Thank you so much for very important comments.

I have added “HTLV-1-associated myelopathy-tropical spastic paraparesis (HAM/TSP), an unremitting and progressive neurological disorder that presents with spastic paraparesis, neurogenic bladder, sphincter dysfunction, and mild sensory disturbance in the lower extremities.” in the author summary (Page 5, line 77-80) and the introduction (Page 7, line 114-118).

4. Line 123 countries which screen – please provide a reference.

　 Response）I added the references for blood donor screening.

Australia, Canada, Japan, Europe (Tynell E, Andersson S, Lithander E, et al. Screening for human T cell leukemia/lymphoma virus among blood donors in Sweden: cost effectiveness analysis. BMJ. 1998;316:1417-1422.)

Brazil (Soares BC, Proietti AB, Proietti FA; Interdisciplinary HTLV-I/II Research Group. HTLV-I/II and blood donors: determinants associated with seropositivity in a low-risk population. Rev Saude Publica. 2003;37:470-476.)

the Dominican Republic （ Eusebio-Ponce E, Candel FJ, Paulino-Ramirez R, Serrano-García I, Anguita E. Seroprevalence and Trends of HTLV-1/2 among Blood Donors of Santo Domingo, Dominican Republic, 2012-2017. Rev Esp Quimioter. 2021;34(1):44-50.）

The United Kingdom (Tosswill JH, Taylor GP, Tedder RS, Mortimer PP. HTLV-I/II associated disease in England and Wales, 1993-7: retrospective review of serology requests. BMJ. 2000;320:611-612. )

Latin America (Gotuzzo E. Risk of transfusion-transmitted human T-cell lymphotropic virus-type I in Latin America. Int J Infect Dis. 2000;4:59-61.)

I deleted two words, “organ donors” and “tissue transplants” and revised the sentence, “HTLV-1 antibody screening for blood donors has been implemented in Australia, Canada, Brazil, Japan, the United Kingdom, Latin America, the Caribbean, and Europe to reduce the risk of HTLV-1 infection through blood product transfusions.” in the introduction (Page 7, line 130-133).

Thank you so much.

5. Line 365 – How do you know that the proposed large scale education campaign will be effective in preventing sexual transmission? Perhaps compare to HIV campaign and reference.

Response) Thank you so much for the important comments. I revised the sentence, “A large-scale educational campaign that includes condom use and avoidance of high-risk sexual behaviors would be effective in preventing sexual transmission of HTLV-1 just as an HIV campaign has been effective.” (Page 31, line 375-378.)

I added two references.

Ref. 43. Frey K, Lociciro S, Blank P, et al. 'Break the Chains 2015' community-based HIV prevention campaign for men who have sex with men in Switzerland: non-randomised evaluation and cost analysis. BMJ Open. 2020;10:e032459.

44. Onoya D, Mohlabane N, Maduna V, van Zyl J, Sewpaul R, Naidoo Y. Testing in the HIV Counselling and Testing (HCT) campaign, HIV risk behaviours and ART enrolment in South Africa. Public Health. 2016;136:152-160.

6. The term “we” is used in the manuscript but there is only one author. In which case either use I or write in the third person.

Response) Thank you very much. In accordance with your comments, I have corrected the term "we" in the original manuscript.

Reviewer #2: 1. As this paper is written, it appears to have an overwhelmingly major flaw: It seems to assume that prevention of an HTLV-1 infection increases the utility of the person in question in by far most cases from 0.712 to 1. The author appears to assume that a person who is not an HTLV-1 carrier, is in perfect health and has a utility of 1. That assumption is incorrect because there are many other factors and conditions than HTLV-1 that can cause less than perfect health. Correction would greatly reduce the number of QALYs gained by screening, possibly by a factor greater than 10, so that cost-effectiveness of screening would change from well within what is generally considered cost-effective, to well outside of that. Correction would therefore reverse the main conclusion of the paper and require a major revision of the text and tables.

Response) Thank you very much for the important comments. I understand what the reviewer is saying. Health utility value of the HTLV-1 uninfected state was estimated to be 1 based on the Japanese EQ-5D-5L value set. To address this issue, I have added a sensitivity analysis for the health utility value of HTLV-1 uninfected status with a range from 0.7 to 1. The sensitivity analysis showed that HTLV-1 antenatal screening is cost-effective at a WTP threshold of US$50,000 per QALY gained when the health utility value of HTLV-1 uninfected state is greater than 0.95.

Ref. Ikeda S, Shiroiwa T, Igarashi A, Noto S, Fukuda T, Saito S, et al. Developing a Japanese version of the EQ-5D-5L value set. J. Natl. Inst. Public Health. 2015;64(1):47-55.

Some comments of less importance than the above:

8. The author mentions several times “more cost-effective” while the paper only compares screening with not-screening, therefore there is only one cost-effectiveness ratio at play, so that there is no comparison (“more”) of cost-effectiveness ratios possible.

Response）Thank you very much for the significant comments. I have deleted “more” in the manuscript.

9. The text seems often unclear about whether a number mentioned concerns incidence or prevalence. E.g. line 78 seems to describe prevalence, while line 111 seems to quote the same number but describes it as incidence.

Response）Thank you very much for the significant comments. All text has been unified with “prevalence”, not “incidence”.

10. At line 155: are those individuals all pregnant women? If so, please say so.

Response) No, those individuals are not all pregnant women. They are 30-year-old individuals born to mothers who did or did not receive HTLV-1 antenatal screening.

11. At line 171: as I read the paper, compliance rate does not affect the estimated cost-effectiveness ratio.

Response) Thank you very much. Yes, the compliance rate does not affect the estimated cost-effectiveness ratio. I understood and deleted the sentence, “The compliance rate for HTLV-1 antenatal screening is assumed to be 100% in the model.”. (Page 11, line 191-193)

12. At line 191 under the heading ‘no screening’: is the probability of sexual transmission not considered in the screening arm?

Response) No, the probability of sexual transmission for HTLV-1 is considered for both HTLV-1 screening and no screening arms. I have added the sentence, “The probability of HTLV-1 transmission through sexual intercourse is considered.” in both the screening arm (Page 12, line 205) and no screening arm (Page 12, line 213-214).

13. It is generally left unclear how sexual transmission affects the cost-effectiveness of screening. Does it have any influence on the presented estimates?

Response) The sensitivity analysis showed that cost-effectiveness was not sensitive to the probability of sexual transmission for HTLV-1. It means that sexual transmission doesn’t affect the cost-effectiveness of screening. I added the sentence, “Sexual transmission rate didn’t affect the cost-effectiveness of screening.” (Page 21, line 326-327) in the results.

14. Does direct transmission play any role in this model?

Response) No, this model doesn’t consider direct contact transmission through blood.

15. The one-way sensitivity analysis does not seem to just involve changing one parameter at the time (with results usually presented in a tornado diagram), but to involve only one parameter altogether.

Response) Thank you very much for the important comments. I deleted the figures of the one-way sensitivity analyses and added the ICER tornado diagram to the figure (Figure 2A). The sentence, “ICER tornado diagram showed that HTLV-1 antenatal screening is cost-effective compared with no screening at a WTP threshold of US$50,000 per QALY gained when the maternal HTLV-1 seropositivity rate is greater than 0.0022, the proportion of long-term breast-feeding is greater than 0.16, the proportion of short-term breast-feeding is greater than 0.04, the proportion of bottle-feeding is lower than 0.04, the health utility value of HTLV-1 carriers is lower than 0.77, the health utility value of HTLV-1 uninfected state is greater than 0.95, and the cost of HTLV-1 antibody test is lower than US$94.7.” (Page 21, line 318-326) was added in the results and Figure 2A.

16. At line 207: Reference 20 does not describe test specificity as 100%. Furthermore, the false positivity rates mentioned there, would cause that around half of test positives are false positives.

Response)　I’m sorry. Thank you very much for the important comment. I deleted the sentence, “The values for sensitivity and specificity of the HTLV-1 antibody test were derived from the literature and were set at 100% [20].” (Page 13, line 234-235) Reference 20 was also deleted.

17. At line 336: This paper does not demonstrate that screening reduces morbidity and mortality but rather, makes literature based quantitative assumptions concerning effects on morbidity and mortality in order to estimate cost-effectiveness.

Response)　Thank you very much for the significant comments. I revised the sentence, “HTLV-1 antenatal screening is cost-effective and reduces ATL and HAM/TSP morbidity and mortality in Japan.” to “HTLV-1 antenatal screening is cost-effective and has the potential to reduce ATL and HAM/TSP morbidity and mortality in Japan.” in the abstract (Page 4, line 69-70), in the author summary (Page 6, line 96-98), and in the conclusion (Page 34, line 437-439).

18. At line 379: The paper leaves unclear how a gender difference in horizontal transmission affects the cost-effectiveness of screening.

Response) Thank you very much. The gender difference of the target populations is not considered in this study, so gender differences in horizontal transmission are not included in the analysis. I used the average of horizontal transmission rates for men and women in the analysis. I added the sentence, “Third, gender differences in HTLV-1 horizontal transmission rates were not taken into account in the model. The HTLV-1 horizontal transmission rate from males to females is greater than the HTLV-1 horizontal transmission rate from females to males.” in the limitations. (Page 33, line 417-420)

19. At lines 386-389: As mentioned under 1., the paper seems to assume that HTLV-1 carriers have a much lower utility than non-carriers therefore it seems incorrect to state that other diseases than ATL and HAM/TSP were not taken into account, on the contrary, it seems that their effects are gravely overestimated.

Response) As noted in the reviewer's comment, HTLV-1 carriers have a much lower utility than non-carriers in this model. However, the health utility of HTLV-1 carriers is set at 0.712 based on the literature (Rosadas C, Assone T, Yamashita M, Adonis A, Puccioni-Sohler M, Santos M, et al. Health state utility values in people living with HTLV-1 and in patients with HAM/TSP: The impact of a neglected disease on the quality of life. PLoS Negl Trop Dis. 2020;14:e0008761). Rosadas et al determined these values for HTLV-1 asymptomatic carriers (0.7121) and HAM/TSP patients (0.2991) using a self-administered questionnaire in patients from Brazil and UK. In their study, most asymptomatic carriers reported some problems in the pain/discomfort domain. The pain prevalence among HTLV-1 infected individuals varies from 35–88% and is more commonly reported as persistent.

I understood the reviewer’s comment and deleted the sentence, “Seventh, I didn’t consider other HTLV-1-associated diseases, such as HTLV-1-associated uveitis, infective dermatitis, bronchiectasis, bronchitis, bronchiolitis, seborrheic dermatitis, Sjögren’s syndrome, rheumatoid arthritis, fibromyalgia, and ulcerative colitis.” from the limitations (Page 33-34, line 426-431) to the discussion (Page 32, line 401-405).

I also performed a sensitivity analysis for both health utilities with a wide range and found that HTLV-1 antenatal screening is cost-effective compared with no screening at a WTP threshold of US$50,000 per QALY gained when ----- the health utility value of HTLV-1 carriers is lower than 0.77 and the health utility value of HTLV-1 uninfected state is greater than 0.95.”. (Page 25, line 307-309)
