# Supplementary material for: Cost-effectiveness of human T-cell leukemia virus type 1 (HTLV-1) antenatal screening for prevention of mother-to-child transmission
Source: PLoS Negl Trop Dis. 2023 Feb 21;17(2):e0011129. doi: 10.1371/journal.pntd.0011129 (PMC9983854; doi:10.1371/journal.pntd.0011129)
Supplement: Attachment — Submitted filename: Ver2 Response to Reviewers comments.docx [file pntd.0011129.s002.docx]

Responses to the reviewer’s comments

Thank you very much for the reviewer’s very important comments. According to very important comments, I completely revised my paper point by point and highlighted my responses to the reviewers’ comments in red. I have written the page and line numbers of the highlighted version of the text to indicate where changes were made.

Thank you so much.

Sincerely yours,

Akiko Kowada, MD, PhD

Reviewer 1:

Line 131 – are 4000 infections in adults and adolescents diagnosed each year or is this the estimated number of new infections per year?

Response)　Thank you very much for the very important comment. This is the estimated annual number of new HTLV-1 infections. Thank you very much for providing this latest reference (Sagara Y, Nakamura H, Satake M, Watanabe T, Hamaguchi I. Increasing horizontal transmission of human T-cell leukemia virus type 1 in adolescents and young adults in Japan. J Clin Virol. 2022;157:105324.)

I revised the sentence based on this latest paper, “More than 2,800 new cases of HTLV-1 infection are estimated annually in Japan.” (Page 7, Line 124-125)

Line 217 and Table 1– the horizontal infection rate is 0.000046 on the 2016 paper of HTLV-1 incidence in Japanese blood donors. This is the infection rate but not the transmission rate. A carrier infected in early life will have potentially more opportunity to transmit than a carrier who does not become infected until they become sexually active. Please also confirm that the transmission rate when incorporated into the model describes the life-time risk of transmission following early life infection and not the risk per 100,000 person years. This is not clear at the moment. I note too that the work of Satake et al has recently been updated in J Clin Virol 2022.
(The incidence density was significantly higher in women (6·88 per 100 000 person-years; 95% CI 6·17-7·66) than in men (2·29 per 100 000 person-years; 95% CI 1·99-2·62; p<0·0001).
The number of seroconversions per 100,000 person-years was 1.54 for men and 4.21 for women – satake et al J Clin Virol . 2022 Dec;157:105324.  doi: 0.1016/j.jcv.2022.105324.
Although the seroconversions per 100,000 person years is lower than in the 2016 report the sero-conversion rate amongst adolescents and Young adults had increased.

Response) Thank you very much for letting me know about the latest literature (Sagara Y, Nakamura H, Satake M, Watanabe T, Hamaguchi I. Increasing horizontal transmission of human T-cell leukemia virus type 1 in adolescents and young adults in Japan. J Clin Virol. 2022;157:105324.) I replaced old literature (Satake M, Iwanaga M, Sagara Y, Watanabe T, Okuma K, Hamaguchi I. Incidence of human T-lymphotropic virus 1 infection in adolescent and adult blood donors in Japan: a nationwide retrospective cohort analysis. Lancet Infect Dis. 2016;16:1246-54.) with this new literature.

HTLV-1 infection rates in the work of Satake et al are not the same as horizontal transmission rates. However, Sagara Y et al obtained the current status of HTLV-1 horizontal infection and calculated the number of seroconversions per 100,000 person-years in adolescents and young adults. They suggested that the obtained annual estimates of the HTLV-1 seroconversion rates might be underestimated and that there is potential for more horizontal HTLV-1 transmission cases. I re-conducted a cost-effectiveness analysis using the new estimated horizontal transmission rate (0.000029) with a wide range of the sensitivity analysis (0.00001-0.00006). I also added the sentence, “Eighth, the horizontal transmission rate was estimated based on the HTLV-1 seroconversion rate among adolescents and young adults [16].” in the limitations. (Page 32-33, Line 433-434)

I confirmed that the life-time risk of the vertical transmission rate that a carrier infected in early life was considered in the model as well as the horizontal transmission rate.

Line 307 – “The sexual transmission rate didn’t affect the cost-effectiveness of screening”. Do you mean that the number of transmissions attributed to early life infection was so low that if did not increase the cost-effectiveness of screening. How many sexual transmissions would have been prevented by the avoidance of mother-to-child transmission. This should be added to the section on cumulative lifetime health effects.

Response) Thank you very much for the very important comments. “The sexual transmission rate didn’t affect the cost-effectiveness of screening”. This means that cost-effectiveness is not sensitive to the horizontal transmission rate of HTLV-1 infection.”. So, I revised it. (Page 20, Line 303-304)

To clarify how many sexual transmissions would have been prevented by the avoidance of mother-to-child transmission, I calculated the cumulative lifetime number of HTLV-1 carriers separately from those due to the vertical and horizontal transmission of HTLV-1 (Table 3). The results showed that the cumulative lifetime number of HTLV-1 carriers with horizontal transmission is higher than those of HTLV-1 carriers with vertical transmission and the cumulative lifetime number of HTLV-1 carriers with horizontal transmission shows little difference between HTLV-1 antenatal screening and no screening. Among HTLV-1 carriers in Japan, 8.51% of those who received HTLV-1 screening and 30.98% of those who did not receive screening are expected to be related to mother-to-child transmission of HTLV-1. (Page 25-26, Line 337-342)

In this section the estimate on ATL-associated deaths is 64% of the total of ATL cases whereas the data input was that the 4 year survival of 16%. Why is the mortality rate in the model so low.  On the other hand 71 HAM associated deaths were prevented out of 177 cases – a 40% mortality. The suggestion in the text is that these numbers may be wrong. What is their contribution to the cost-effectiveness?

Response) To answer this question, I first recalculated the cumulative lifetime ATL- and HAM/TSP-associated deaths by inputting the new horizontal transmission rate (0.000029) into the model. I must explain the calculation methods. There are five types of ATL with different 4-year survival rates (16.8% to 62.1%). In the model, mortality due to other diseases is also considered. By the Markov cohort analysis, I calculated the cumulative lifetime ATL cases and ATL-associated deaths as the sum of five types of ATL occurring between the ages of 30 and 100 years. HAM/TSP cases and HAM/TSP-associated deaths are also recalculated in the new model with the new horizontal transmission rate (0.000029) for the 70-year cumulation. The results showed that the cumulative lifetime ATL-associated deaths are 65% of the total of the cumulative lifetime ATL cases and the cumulative lifetime HAM/TSP-associated deaths are 36-37% of the total of the cumulative lifetime HAM/TSP cases.　I checked the model again and again and verified that these numbers are accurate and the results are correct (Table 3).

In the response to Reviewer one you state Yes, the association of ATL risk with acquisition of HTLV-1 in early life was considered in the analysis. In the model, HTLV-1 is transmitted mainly through mother-to-child transmission (MTCT), even if horizontal transmission through sexual intercourse is being considered. However, the data used for ATL risk was the published incidence rate – which would include all persons infected with HTLV-1 regardless of the age of infection. Whilst ATL has been reported following infection in adult life a strong association with infection in early life remains. The relative contribution of infection in early v adult life is therefore likely to impact significantly in the model. What proportion of all HTLV-1 infection in adults in Japan did you consider to be related to mother-to-child transmission? The increasing rates of infection with increasing age, especially among females, indicate a considerable infection after early life.
Response) Thank you very much for the very important comments. To clarify the proportion of all HTLV-1 infections in adults in Japan that are related to mother-to-child transmission, I calculated the cumulative lifetime numbers of HTLV-1 carriers separately with the vertical and horizontal transmission at HTLV-1 antenatal screening and no screening by the Markov cohort analysis. The cumulative lifetime number of HTLV-1 carriers with horizontal transmission is higher than the cumulative lifetime number of HTLV-1 carriers with vertical transmission. Among HTLV-1 carriers in Japan, 8.51% of those who received HTLV-1 screening and 30.98% of those who did not receive screening are expected to be related to the mother-to-child transmission of HTLV-1 (Table 3). (Page 25-26, Line 337-342)

In your response to point 16 Reviewer 1 you have stated that anxiety related to screening would be a particular feature of HTLV-1 screening. It is not clear that this was your intention.  If not, please amend. If so, why for example would HTLV-1 screening cause more anxiety than other antenatal screening tests – eg  for Down’s syndrome or HIV?
Response) Thank you very much. This was not my intention. I deleted the sentence, “All diagnostic tests and screening programmes are inevitably associated with a degree of anxiety. This would be particularly a feature of HTLV-1 antenatal screening. Depression and anxiety would occur when parents discover that they were not offered testing and interventions for a preventable infection causing cancers and progressive neurological diseases and see these diseases occurring in their children even as adults. When introducing HTLV-1 antenatal screening, it is necessary to consider an enhanced counseling system to minimize the negative psychological aspects of HTLV-1 carriers. In addition, other HTLV-1-associated diseases including HTLV-1-associated uveitis, infective dermatitis, bronchiectasis, bronchitis, bronchiolitis, seborrheic dermatitis, Sjögren’s syndrome, rheumatoid arthritis, fibromyalgia, and ulcerative colitis may appear in HTLV-1 carriers and may enhance depression and anxiety.”. I added the sentence in the limitations, “Ninth, the model did not account for other HTLV-1-associated diseases such as HTLV-1-associated uveitis, infective dermatitis, bronchiectasis, bronchitis, bronchiolitis, seborrheic dermatitis, Sjögren’s syndrome, rheumatoid arthritis, fibromyalgia, and ulcerative colitis.” (Page 32-33, Line 434-438)
