# Supplementary material for: Cost-effectiveness of human T-cell leukemia virus type 1 (HTLV-1) antenatal screening for prevention of mother-to-child transmission
Source: PLoS Negl Trop Dis. 2023 Feb 21;17(2):e0011129. doi: 10.1371/journal.pntd.0011129 (PMC9983854; doi:10.1371/journal.pntd.0011129)
Supplement: Attachment — Submitted filename: Ver3 Response to Reviewers comments.docx [file pntd.0011129.s003.docx]

Responses to the reviewer’s comments

Thank you very much for the reviewer’s very important comments and great suggestions. According to the reviewer’s comments, I completely revised my paper point by point, modified the model, and highlighted my responses to the reviewer’s comments in red. I have written the page and line numbers of the highlighted version of the text to indicate where changes were made.

I deeply appreciate the reviewers’ great suggestions.

Sincerely yours,

Akiko Kowada, MD, PhD

Reviewer #1: Line 166-167 author say that a hypothetical cohort of 30-year individuals was targeted. Is the author referring to pregnant women? The model should focus on pregnant women, since they are the target population of the intervention. This needs to be clear.

Response) Thank you very much for the important comment. I needed to explain in more details why I am targeting babies born to the mothers who have had or not had received HTLV-1 antenatal screening. Withholding breastfeeding from mothers with positive results of HTLV-1 antenatal screening is an effective strategy to prevent future ATL for their babies. Therefore, I focused on the prevention of vertical transmission of HTLV-1 infection from mother to baby in this cost-effectiveness study and targeted individuals who have grown up from babies and are now 30 years old.

I revised the sentence, “A hypothetical cohort of 30-year-old individuals born to mothers who had or had not received HTLV-1 antenatal screening was targeted from a healthcare payer perspective with a lifetime horizon.”. (Page 10, line 170-171)

Line 191 “Patients with favorable and smoldering types of ATL continue to be followed up without treatment”. How long are they followed up for? What rate of transformation to the more aggressive forms of ATL were imputed in the model.

Response)　Thank you very much for very important comments. I have modified the model by adding transformation rate from favorable chronic-type and smoldering-type ATL to acute-type ATL, and the mortality of favorable chronic-type and smoldering-type ATL. (Page 11, line 196-199) (Page13, line 222-223)

In the addition, two new references were added to the references section.

References

26) Takasaki Y, Iwanaga M, Imaizumi Y, et al. Long-term study of indolent adult T-cell leukemia-lymphoma. Blood. 2010;115(22):4337-4343. doi:10.1182/blood-2009-09-242347

27) Taniguchi H, Imaizumi Y, Takasaki Y, et al. Clinical features at transformation in adult T-cell leukemia-lymphoma with smoldering and chronic types. Int J Hematol. 2019;109(4):402-408. doi:10.1007/s12185-019-02602-4

line 194-195 Author states "The probability of HTLV-1 transmission through sexual intercourse is considered", does it refer to the sexual transmission in the 30-year-old cohort (from sexual partners to pregnant women or from pregnant women to their sexual partners?) or the sexual transmission that may occur during the lifetime of the babies born from those mothers? If it refers to the 30-year-old cohort, it is not expected that sexual transmission would affect or be affected by antenatal screening program and should not be considered. However, prevention of sexual transmission to a partner can also be prevented by safer sex (condom use), once the women is aware of their serostatus due to antenatal screening.

If it refers to secondary sexual transmission throughout the babies` life author should not use the incidence rate in blood donors. The horizontal transmission rate used is that of the incidence found among persons of reproductive age however all models of infection have shown the prevalence of infection to increase with age and that the rate of increase is higher in older age. Therefore the model should not use the same horizontal transmission rate throughout the person’s life as this under-estimates the number of infections prevented.

The transmission rate of HTLV through sex is expected to be much higher than incidence in blood donors at reproductive age, but would vary according to different variables, such as gender of the infected individual, number of partners, frequency of unprotected sex, co-infection with other STI, HTLV proviral load, etc. The rate of sexual transmission was estimated as 60% from male to female and 0.4% from female to male in a 10y period, but this is an estimation only and has a lot of uncertainties. In the Miyazaki cohort of sero-discordant couples the transmission rate over 5 years was 7% with a male-to-female rate 4-fold higher than females-to-males. Some references regarding sexual transmission rate

https://pubmed.ncbi.nlm.nih.gov/15809908/

https://pubmed.ncbi.nlm.nih.gov/8418183/

https://pubmed.ncbi.nlm.nih.gov/2877031/

In terms of those who are infected in early life – there is clearly a longer period of risk of horizontal transmission that those who are infected in later life, but we do not know whether this translates into a greater overall risk of horizontal transmission as behavioural factors may differ between the two risk groups.

If horizontal transmision is being considered the model should include the transmissions relating to the unscreened pregnant lady and transmissions relating to the infants who become infected as a result of the non-screening strategy. As there is only one row for horizontal transmission it seems that only the mother is considered but this is not completely clear.

Response) Thank you very much for the reviewer’s very valuable suggestions and the references. I have excluded this unclear horizontal transmission rate of HTLV-1 from the model, modified the model, and recalculated all results. (Page 11, line 201-202) (Page 12, line 208-209) (Page 13, line 227-228)

Reviewer #1: Line 304-305 "Cost effectiveness is not sensitive to the horizontal sexual transmission rate". See comment above.

Response) I deleted this sentence. (Page 21-22, line 324-325)

Table 3. the cumulative lifetime HTLV-1 carriers with horizontal transmission is higher if antenatal screening is implemented, with a gain of 91 cases. This does not seem to be correct. Higher number of infected people is expected without antenatal screening, and therefore, more people would be able to transmit HTLV through sex. There is no reason that could explain higher number of sexual transmission if antenatal screening is implemented.

Response) Thank you very much for the important comments. I agree with the reviewer. I have excluded unclear horizontal transmission rate from the model and reconstructed the model without horizontal transmission rate of HTLV-1.

Table 3. ATL cases are primarily associated with vertical transmission. Therefore, the reduction of number of ATL cases should be proportional to the reduction of number of infections that would be prevented. In table 3 number of carriers with vertical transmission reduces from 158,244 to 32,822, while number of ATL reduces from 14,804 to 10,392. Even considering horizontal transmission, the number of ATL seem to be too high. Please explain the model for generating the number of cases of ATL. It would appear from the numbers that the model considered most cases of ATL to be consequence of horizontal transmission.

Response) I excluded the HTLV-1 horizontal infection rate from the model according to the reviewer’s suggestions and recalculated the numbers of HTLV-1 carriers and ATL cases. Please note that these numbers are calculated using a Markov model and accumulated over a lifetime. The numbers of HTLV-1 carriers with vertical transmission reduces from 158,244 to 32,822, and the numbers of ATL cases reduces from 5,558 to 3,830.

Table 3 – the LY gain is 456 despite the 4412 fewer cases of ATL – this amounts about 1 month of life expectancy gain for each case of ATL prevented – again this seems difficult to explain.

Response) I modified the model and recalculated the cumulative lifetime LYs gain, 631 LYs, and the cumulative lifetime ATL reduction, 4405. Outcomes were calculated over a lifetime. Lifetime horizon means 70 years from 30-years-old to 100-years-old, but the calculated lifetime LYs per person with HTLV-1 antenatal screening and no screening is 24.94813 LYs and 24.94807 LYs. These results reflect the poor prognosis of ATL. The lifetime LYs gain of HTLV-1 antenatal screening compared with no screening is only 0.0006 LYs. For 10,517,942 babies who were born between 2011 and 2021, the cumulative LYs gain is only 631. Therefore, it could explain the small difference in cumulative lifetime LYs gain between the two strategies. The LYs gain is calculated based on the Markov model and accumulated over a lifetime. Please focus on the cumulative QALYs gain, the cumulative lifetime number of HTLV-1 carriers, ATL and HAM/TSP cases, and ATL and HAM/TSP deaths.

Reviewer #1: The study addresses an important gap in the literature. It is not clear, however, how horizontal transmission was considered in the model. This is a major limitation and should be addressed.

Response) Thank you very much for the reviewer's major point about horizontal transmission of HTLV-1. The reviewer suggested that there is no actual value that accurately reflects the horizontal transmission rate of HTLV-1 infection. I excluded horizontal transmission rate of HTLV-1 infection from the model and recalculated all results.
